# DETERMINANTS AND CONSEQUENCES OF LEISURE ACTIVITY ENGAGEMENT AND CONSTRAINTS IN OLDER ADULT POPULATIONS

**DOI:** 10.1093/geroni/igac059.800

**Published:** 2022-12-20

**Authors:** Angie Sardina, Alyssa Gamaldo, Alyssa Gamaldo

## Abstract

Older adults engage in approximately 7.1 hours/day in leisure activities; however, much of the leisure activity engagement comprises of passive activity engagement (e.g., watching TV). An increasing amount of literature suggests that regular engagement in cognitively and physically stimulating activities, rather than strictly passive activity engagement, is associated with better physical and mental health as well as maintenance of social networks. As the aging population continues to increase and levels and types of activity engagement are shifting across our more diverse older adult populations, it is imperative to understand levels and types of activities older adults are participating in, as well as the psychosocial and contextual factors related to leisure activity engagement. This symposium will include presentations from studies that explore the following: (1) leisure activity interests, engagement, and constraints; and (2) determinants and/or consequences of leisure activity engagement. Specifically, Sardina and colleagues examined daily variability between affect and leisure engagement, and explored potential sociodemographic moderators for these associations. Tian and colleagues explored the association between leisure activities and modes of transportation. Tian and colleagues explored leisure activity engagement with prospective daily diary methods, and examined associations between leisure activities and physical health. Janke and colleagues explored associations between facilitators, constraints, and constraint negotiation and self-reported physical activity levels for older adults with arthritis.

